# The perceived affordances of simulation-based learning: online student teachers’ perspectives

**DOI:** 10.1186/s41239-022-00366-2

**Published:** 2022-12-19

**Authors:** Lynn Dittrich, Toril Aagaard, Hjørdis Hjukse

**Affiliations:** 1 Department of Educational Science, University of South-Eastern Norway, 3679 Notodden, Norway; 2 Faculty of Humanities, Sports and Educational Science, University of South-Eastern Norway, 3679 Notodden, Norway

**Keywords:** Simulation-based learning, Online students, Pre-service teachers, Mediating professional learning, artefacts, Peer-led focus groups

## Abstract

In this study, we explore the perceived affordances of simulation-based learning (SBL) among online student teachers in Norway. We investigated how simulations mediate professional learning when coupled with educator-led debriefings and peer-led focus groups. Using an exploratory qualitative design, we examined 21 pre-service teachers’ perspectives on their experiences of this SBL intervention. We analysed peer-led focus group data, as well as written reflection logs. Our findings reveal that despite experienced technical challenges, the overall intervention afforded work-life relevant practice, prompting collaborative reflection. In addition to expert feedback, the peer-led focus group discussions are found to be a key component of the SBL design, as these enabled collaborative reflection over a shared practice teaching experience. The findings imply that SBL can be used to bridge the theory-practice gap, a recurring challenge in the field. Consequently, SBL has transformative potential in teacher education.

**Supplementary Information:**

The online version contains supplementary material available at 10.1186/s41239-022-00366-2.

## Introduction

There is an increasing body of knowledge on simulation-based learning (SBL), spanning various professionally oriented disciplines. Across the different professions, SBL is said to provide students opportunities to develop a wide array of skills and to engage in more active forms of learning in higher education (Hallinger & Wang, 2020). Simulation technologies are increasingly being used to bridge the theory-practice gap (Hopwood et al., 2016) and have been suggested as useful for providing practical and experiential learning before practicum experiences (Fischetti et al., 2021). Still, little is known about the perceived affordances of SBL, particularly from the perspectives of online student teachers. Furthermore, given the growing number of online teacher education programmes globally (Dyment & Downing, 2020) and the impact of Covid-19 on the practicum (Bondie et al., 2021; Walters et al., 2021), more research on online students’ perspectives of SBL is warranted. Previous studies call for future research to explore how online or distance students might benefit from SBL (Hudson et al., 2018; Spencer et al., 2019). Recent studies indicate that simulations can be used to engage students in more practice training both in virtual and in-person contexts (Bondie et al., 2021).

A persistent problem both internationally and in Norway has been the attrition of teachers (Christophersen et al., 2016). In Norway, up to 33% of new teachers leave the profession within five years of completing their studies (Tiplic et al., 2015). In a recent study about new teachers’ job perceptions, mastering classroom management skills was directly correlated to higher job satisfaction and lower stress (Brandmo & Tiplic, 2021). Moreover, because university programmes are perceived as theoretically focused, simulation technologies are a potential solution for increased practice in teacher education. Kaufman & Ireland (2016) recommend simulations be considered in teacher education to “augment practicum experience with a cycle of practice, feedback, reflection and repeated practice” (p. 264). Simulations can thus strengthen and supplement the practicum experience by offering students opportunities to apply theoretical knowledge prior to practice in schools. Against this backdrop, we examine online student teachers’ perspectives of SBL based on their initial experience with using TeachLivE™, a mixed-reality classroom simulation. These students mostly have synchronous online lessons and meet on campus a few weeks every year. Their field placements are spread across Norwegian schools. Consequently, they seldom have shared practice teaching experiences with peers. Developed at the University of Central Florida and commercialised by Mursion, TeachLivE™ is a simulated classroom, providing pre-service teachers opportunities to practice various teaching skills. It is currently being used by over 80 universities globally (Ersozlu et al., 2021).

Our aim is to examine the potential of simulation technologies for practical and experiential learning opportunities, particularly within online teacher education settings. We address the following research question: What perceived affordances emerge when online student teachers engage in simulation-based learning? By this, we refer to the students ‘perception of the simulation technology as well as the overall learning environment within which it was implemented.

In the following, we discuss related research on SBL, before presenting our theoretical position, followed by our methodology. Thereafter, we present the findings, showing the perceived affordances of SBL from the online students’ perspectives. These are discussed in relation to previous research. We then present our concluding remarks, including suggestions for future research.

## Related research on simulation-based learning

Research on the use of simulations in teacher education is growing and multifaceted. It ranges from research on highly immersive virtual reality simulations (Huang et al., 2010; Lugrin et al., 2016) to simulations that do not make use of technology but involve role-plays with actors (Levin & Flavian, 2020). To contextualise this study and to build on previous research we discuss related studies on SBL within teacher education, paying particular attention to desktop simulations like TeachLivE™.

A simulation can be defined as “a simplified, dynamic and precise representation of reality” (Sauvé et al., 2007, p. 252) which “allows users to encounter problem situations, try decisions and actions, experience the results and modify their behaviour without risking harm (Kaufman & Ireland, 2016, p. 261). Since simulations seek to emulate real situations, previous literature on SBL has focused on fidelity. However, the perception that higher fidelity results in better simulation experiences, has been nuanced and researchers have found that credible scenarios, based on authentic, real-world cases are also of relevance to students’ experience (Rooney et al., 2015). Furthermore, the success of SBL seems to depend on how willing students are to believe that the simulation represents a real-world setting. Consequently, the willingness to suspend disbelief seems to be important for students’ sense of realism in a simulation (Dede, 2009; Dieker et al., 2014). Although TeachLivE™ has been reported as useful for both pedagogical and content-specific practice (Hayes et al., 2013b), it has also been reported that some students find it difficult to completely suspend disbelief (Dalinger et al., 2020).

Another recurring topic in the research on SBL is the importance of debriefings (Bondie et al., 2021; Hallinger & Wang, 2020), identified as the “heart and soul” of simulation-based training (Rall et al., 2000, p. 517). Debriefings are an opportunity to co-construct knowledge after a simulation experience (Battista, 2015). Although debriefings tend to be educator-led, a study by Judge et al., (2013) included student-led feedback after participants had practised behavioural management strategies while teaching avatars. Students were divided into groups, one receiving peer focus group feedback and email feedback, the other not receiving any feedback. The study showed that TeachLivE™ was useful for training students on managing undesirable behaviours while reinforcing positive behaviours. Students who showed the highest improvement in using behaviour management strategies were those who had received peer feedback (Judge et al., 2013). Across studies, feedback and reflection in debriefings are found to be valued and are reported as giving students the opportunity for meaningful coaching and scaffolding (Cohen et al., 2020) as well as helping to build students’ teaching confidence (Dalinger et al., 2020; Khalil et al., 2016). TeachLivE™ has also been reported as improving students teaching self-efficacy beliefs after continued sessions (Bautista & Boone, 2015; Gundel et al., 2019; Samuelsson et al., 2022).

In addition, there is agreement among educational researchers that simulation technologies offer a safe space for students to enact practice and receive constructive feedback on specific skills (Dawson & Lignugaris/Kraft, 2016; Dieker et al., 2014; Judge et al., 2013). Several studies conclude that TeachLivE™ supports training high on leverage practices such as classroom management (Dalinger et al., 2020; Fischetti et al., 2021; Hudson et al., 2019; Judge et al., 2013; Pankowski & Walker, 2016). Still, more knowledge is needed in the teacher education field to understand the potential and pitfalls of SBL (Chernikova et al., 2020). Specifically, Chernikova et al., (2020) find that there is a lack of knowledge on exactly who SBL is helpful for, what scenarios are useful and the role of additional support for learning. While there are studies that shed light on SBL in rural education settings (Hudson et al., 2019), little is known about SBL in online teacher education settings. We add to the knowledge on the perceived affordances of SBL by focusing on online students’ perspectives of both the potentials and challenges experienced. Moreover, our research focuses on aspects that have been limited in previous research, namely a sociocultural perspective, online teacher education and the consideration of post-debriefing social interaction in the SBL design.

## Theoretical framework

The design of this study is informed by Vygotsky’s (1978) sociocultural perspective which recognises the role of mediating artefacts and social interaction in learning processes. Simulation technologies emulate real world experiences and can thus be seen as a specific type of digital artefact which have a mediating role in professional learning (Orland-Barak & Maskit, 2017). Furthermore, digital artefacts like simulation technologies are “human-made for certain purposes within a specific cultural-historical context” (Aagaard & Lund, 2020, p.20). Such purposes may include “supporting communication and intersubjective knowledge building” (Trausan-Matu & Slotta, 2021, p. 552). Here, this makes us aware of both learning processes mediated by using the simulation technology, but also the learning processes supported by social interaction.

The concept of affordances (Gibson, 1977) has previously been used to understand the interplay between the artefacts and their users. Because we are particularly interested in students perspectives, we apply the concept of perceived affordances (Norman, 1999, 2013) in our analysis. Distinguished from Gibson’s (1977) concept of affordances or action possibilities, perceived affordances can be defined as “actions the user perceives to be possible” (Norman, 1999, p. 39) Norman’s (1999; 2013) conceptualisation underscores the importance of the user’s perception of action possibilities and is thus suitable for understanding technology-mediated social learning environments. To this end, Kirschner et al., (2004) propose a framework comprising three elements namely, the educational, social and technological affordances. Educational affordances refer to “relationships between the properties of an educational intervention and the characteristics of the learner” that enable learning (Kirschner et al., 2004, p. 15). Social affordances invite students to take part in learning activities that encourage social interaction and collaboration (Kreijns et al., 2013). Technological affordances refer to aspects pertaining to usability and thus Kirschner et al., (2004) argue that artefacts that are usable but do not invite educational and social engagement are worthless. We use this framework to study the perceived affordances of both the learning environment and TeachLivE™ itself. We thus add knowledge on how the learning environment, both educational and social elements as well as the simulation technology potentially influence culturally rooted, traditional epistemic practices – an area often neglected in the literature (Aagaard & Lund, 2020, p. 20).

## Methodology

In this study, we used an exploratory qualitative design, analysing video recordings of peer-led focus groups and pre-service teachers’ written reflection logs to explore their perspectives of SBL.

### Sampling, participants and the intervention

The SBL design was implemented online using TeachLivE™ via video-recorded Zoom sessions that took place over two days during the 2021 spring semester. Participants were sampled through purposive and convenience sampling (Bryman, 2012) and included 21 fourth-year students enrolled in a 5-year master’s degree programme for initial teacher education (grades 5–10) at a Norwegian university. Participants included 10 males and 11 females, mostly aged between 20 and 30 years. A few were between 30 and 40 years of age. The students were split into 6 groups as indicated in Table 1, based on whether or not they had mathematics as a subject and on their availability.

**Table 1:** Participant details by group

Groups	Focus of instruction	Participants	Pseudonyms
G1	Mathematics didactics	4	Patrick, Irene, Elizabeth, Sophie
G2	Mathematics didactics	4	Brian, Samantha, Pablo, Lisa
G3	Mathematics didactics	3	Alex, Kimberly, Mary
G4	Pedagogy	4	Thomas, Sam, Justin, Matthew
G5	Pedagogy	3	Sarah, Anne, Wendy
G6	Mathematics didactics	3	Kevin, Kate, Mark

One student from each group chose the teacher role, teaching fifth-grade avatar pupils whilst the others observed and prepared feedback (interestingly, in five of the six groups, males chose the teacher role). In addition to training on classroom management, two groups had pedagogy as their core focus, while four groups trained on mathematics didactics. Other observers included one mathematics and one pedagogy educator, and the researchers, one of whom was also the course instructor. Before the intervention, the course instructor briefed the students about the research and the SBL design. She also provided information on the course webpage about the intervention. The students were informed that those having the teacher roles could pause the simulation at any point to get guidance from peers and educators. Furthermore, as part of the briefing students also were given access to a short video clip about the simulation.

TeachLivE™ allows the student teachers to practice teaching to a class of avatar pupils. The avatars have different archetypal personalities, abilities and behaviours. An interactor, who is invisible to the students, controls the avatars’ responses and body language from a different location. This allows real-time interaction. In our case, the interactor is an experienced teacher and trained simulation specialist who uses his expertise to digitally control and speak on behalf of the avatars. He is well-versed on the avatars’ personalities and the scenario. The teaching students were tasked with being substitute teachers. The scenarios had relevance for the students’ learning outcomes, either pedagogy or mathematics. The first avatar they met was the school principal (who is also controlled by the interactor). The principal answered questions that students had about the avatar pupils before entering the virtual classroom. For instance, the principal clarified that the avatars are able to work together in pairs. Groups 4 and 5 led a classroom discussion, planning a fundraising event while the mathematics students taught a lesson on fractions. All groups were faced with classroom management problems and dilemmas. Behaviours displayed by the avatars and enacted by the interactor, included the use of mobile phones, interrupting the teacher, withdrawing or veering off-topic. These sessions were approximately 15 min in duration.

**Fig. 1:**
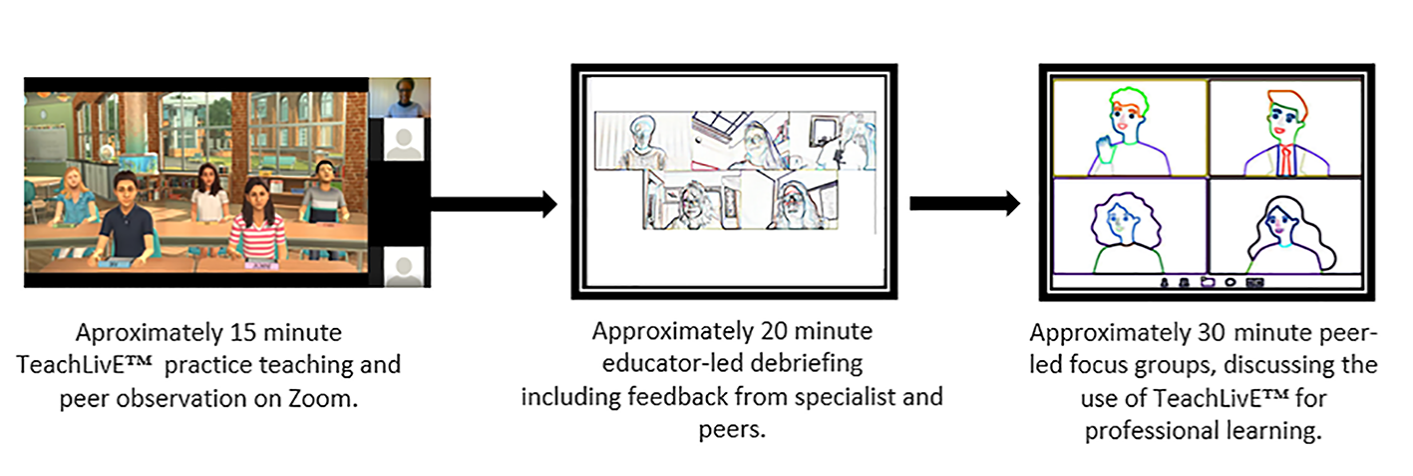
The intervention. Note: Permission to use the image granted by Mursion

In the educator-led debriefing which followed, the simulated classroom activities and interactions were discussed, and the student who had taken the teacher role received feedback from educators and peers. Thereafter, all the students wrote reflection logs about their individual experiences with the simulation. In addition, students participated in peer-led focus groups, in which they collectively reflected upon their experience of SBL with TeachLivE™ for approximately 30 min. The reflection logs and peer-led focus groups were guided by questions eliciting their experience and perceptions of either teaching or observing (Figure 1).

### Data collection

The peer-led focus groups and individual written reflections are the primary data sources^4^ for this study. In recent research, Djohari & Higham (2020) recommend peer-led focus groups as they allow students to “share a dialogic space that facilitates deeper collaborative understanding of their situation. In this context the process itself becomes valuable, not just the data produced” (p. 660). Being online students, the participants were accustomed to having collaborative discussions in online break out rooms in their study programme. Each group was assigned a peer moderator who facilitated the discussions. We designed both closed and open-ended questions^5^ to guide the group discussions and the written reflections. These began with general questions aimed at getting students’ initial impression of the simulation. Thereafter, questions elicited perceptions of the simulation including for example how students experienced the practice as relevant for their learning outcomes. Lastly, we raised questions about the group discussions to elicit meta-reflection.

### Data analysis

In the analysis, we applied the thematic analysis principles by Braun & Clarke (2006) as shown in Table2 below. Soon after the intervention, we watched and listened to the recordings for familiarisation and to begin the pattern-finding process (Derry et al., 2010). We created one content log for each recording, using a table format, in which we included transcriptions and our initial reflections. Content logs include “major events that took place for each brief standard unit of time (e.g., 3 min)”, time-specific markers, and a description of meaningful events in the video data (Derry et al., 2010, p. 18). We also re-read the students’ reflection logs. The first author continued to inductively analyse and code the data from the content logs and reflection logs using Nvivo^6^, resulting in five subthemes. In vivo codes have been used to present these subthemes. The deductive analysis involved using the concept of perceived affordances as conceptualised by Kirschner et al., (2004) to present the three main themes. The data analysis was an iterative process involving reviewing subthemes and themes until consensus was reached. Finally, we revisited the videos and written reflections to verify that the findings identified took both aspects into account, namely representing the data and responding to the research question.

**Table2:**
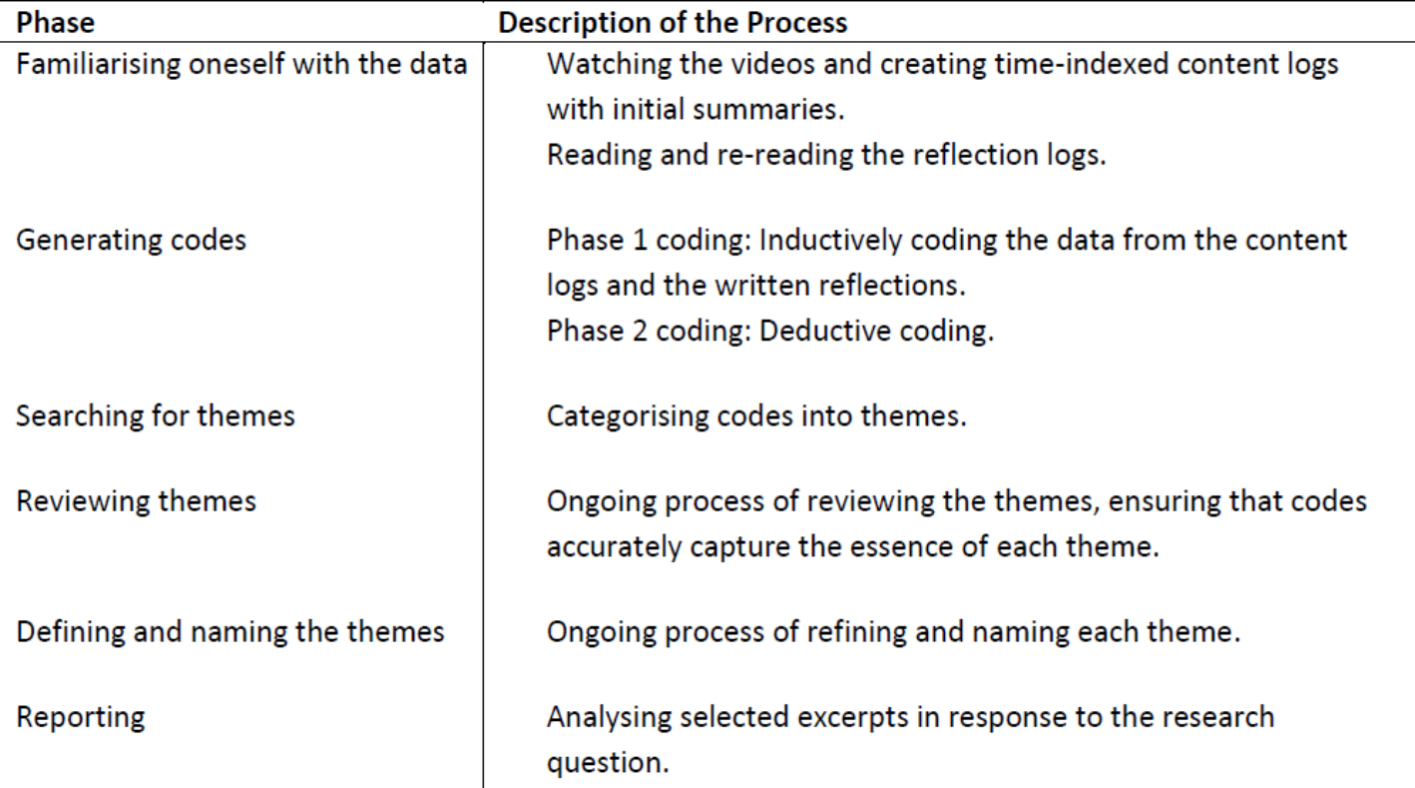
Thematic analysis process. Adapted from Braun & Clarke (2006)

According to Tracy (2010) making use of multiple researcher perspectives and data sources enhances the quality of the findings. In this study, we made use of two data sources. In addition, two of the authors are teacher educator specialists in pedagogy with an insider perspective, while the first author is an educational researcher with a more outsider perspective. These multiple perspectives have helped us in the process of interpreting and refining the themes and subthemes.

## Findings

Through use, artefacts - like simulation technologies - can have transformative potential (Säljö, 2010). However, as Norman (1999) points out, the affordances of an artefact depend on what the user perceives. Drawing from Kirschner et al., (2004) we have conceptualised perceived affordances as comprising educational and social functionality as well as technological affordances. In response to the research question, our analysis revealed three themes and five subthemes as presented in Table3. The themes pertain to the perceived affordances of the learning environment as well as the TeachLivE™ simulation respectively. Below, we present these themes. We discuss these findings thereafter.

**Table3:**
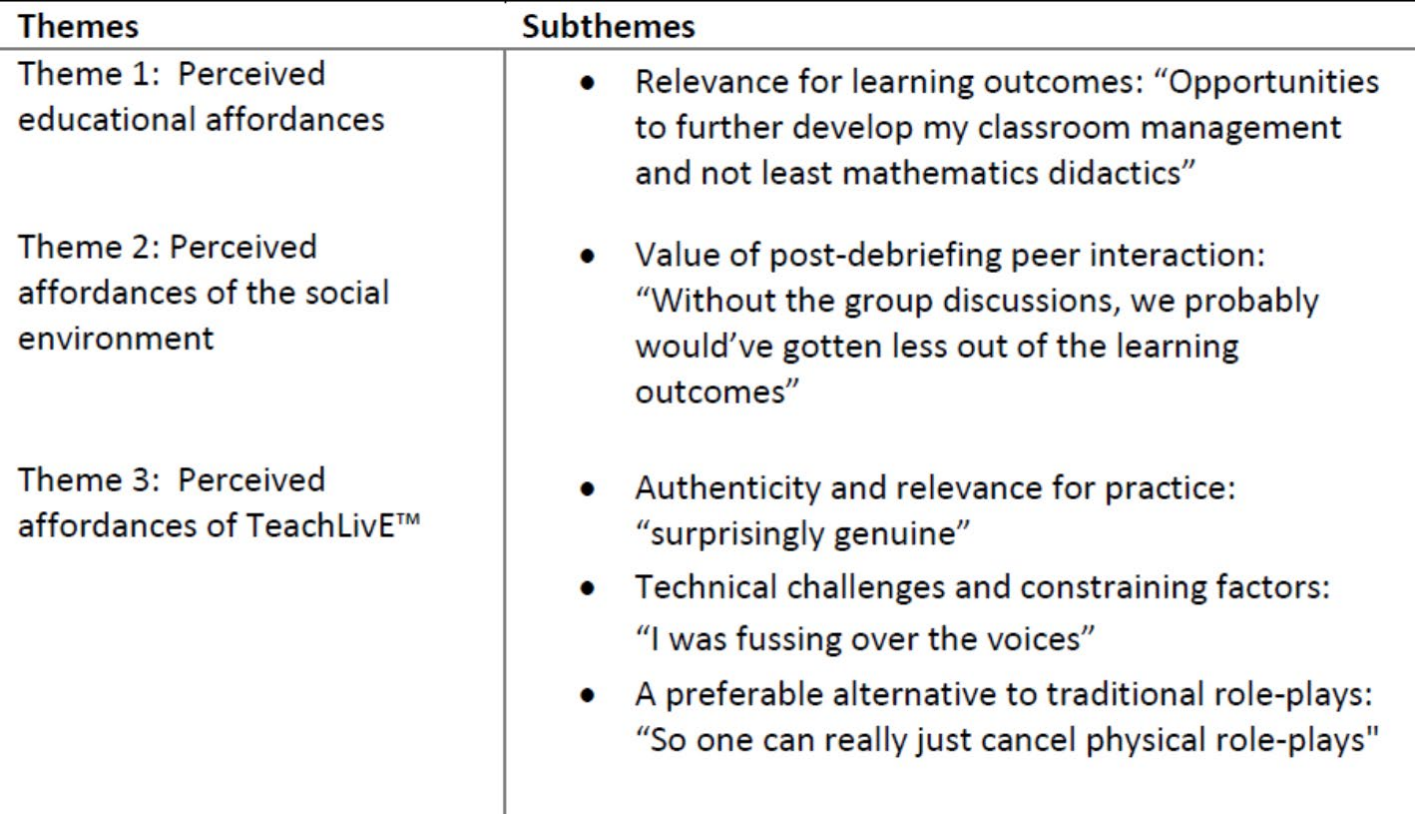
Overview of themes and subthemes

### Theme 1: Perceived educational affordances

#### Relevance for learning outcomes: “Opportunities to further develop my classroom management and not least mathematics didactics”

Generally, students perceived the SBL intervention as a space for practising didactics and classroom management skills. Building and supporting positive relationships with and between the (avatar) pupils, is one example of a learning outcome that was addressed. One observing student discussed how the simulation reinforced her prior knowledge of paying attention to the socio-emotional environment in the classroom:

*Kate, G6: I got how important it is to build relationships with students, that it’s important to spend time on it so that students feel secure, that’s what I think I’ll take further with me. I was aware of it before, but occasionally it becomes easy to forget those who are quiet and a little invisible.*

Students’ classroom management skills were supported as illustrated by one of the students who taught in the simulation, alluding to receiving “*opportunities to further develop my classroom management and not least mathematics didactics*” (Patrick, G1). Similarly, Justin highlighted how the experience helped him reflect on dealing with different pupils, pointing out the challenge of finding suitable ways of creating an inclusive environment:

*Justin, G4: The session has made me reflect more on the focus on classroom management, and different types of students. How to try to include as many people as possible, and that one may need to think extra carefully about the choice of methods of democratic decision-making (…).*

Overall, these quotes imply that students perceived the simulation as enabling opportunities to practice skills like for example creating an inclusive classroom environment. Furthermore, the simulation was perceived by some students as a safe space for trial and error. Sophie for instance perceived the simulation as affording training in a risk-free environment within a learning community:

*Sophie, G1: Getting to test strategies in a setting where you get feedback from students there and then (…) is very educational. These are very safe frames where you don’t have to think about how your reaction will affect a “real” student.*

In sum, we find that the online student teachers perceive that the simulation offered them useful, safe frames for practising teaching and observing peers. In addition, they engaged in giving and receiving feedback, supporting their professional development.

### Theme 2: Perceived affordances of the social environment

#### Value of post-debriefing peer interaction: “Without the group discussions, we probably would’ve gotten less out of the learning outcomes”

During the meta-reflection, students discussed that in addition to appreciating feedback from educators, they also found the peer discussions very meaningful. For instance, one of the students who played the teacher role states that dialogue with peers post-simulation helped *“clarify didactic choices I made”* (Brian, *G2).* Such statements indicate that students benefit from discussing and confirming their strategies and choices with peers. These interactions also helped students reflect upon the simulation experience in relation to their course learning outcomes and allowed for constructive feedback:

*Mary, G3: (….) Without the group discussions, we probably would’ve gotten less out of the learning outcomes.*

*Sam, G4: The most important is talking to others and getting feedback to develop your understanding.*

Kate indicated that the group discussions were “educational” and afforded the opportunity to “reason together”. Furthermore, Sarah succinctly states:

*Sarah, G5: (…) the group discussions are just as valuable to learning as the simulation. Because reflecting and speaking with others is almost more valuable for learning than being in the simulation. But they are linked closely together.*

*Kevin, G6*: *it’s incredibly good to have group discussions afterwards*.

These quotes elucidate the rich learning experience that peer-led focus groups afforded as students had opportunities to interact and discuss problems of practice. The environment, namely the group discussions, enabled reflection and discussion or social interaction. Although the peer-led discussions were organised to gather data to answer the research questions, it is particularly interesting to note that students across the groups consistently valued peer interaction as important. Thus, including the opportunity for post-debriefing peer interaction was perceived as an affordance of the SBL design.

### Theme 3: Perceived affordances of TeachLivE™

#### Authenticity and relevance for practice: “*Surprisingly genuine*”

Although there were mixed reactions regarding the authenticity of the simulated classroom, most students found the experience engaging and realistic. There is evidence in each group of students’ engagement and amazement of the realistic interactions with the avatars. When asked about their initial impression of the simulation, most had positive sentiments, with only 3 out of the 21 students stating in their reflection logs that they did not experience the simulation as realistic. Nonetheless, students like Kimberly explained how the simulation exceeded her expectations:

*Kimberly, G3: I thought the simulation was surprisingly genuine. I thought it was a really nice way to practice. I thought the class and the situations that arose were very credible.*

Some students had initial reservations but soon realised that the interaction in the simulation closely represented real classroom settings:

*Sophie, G1: It was a bit difficult to know how serious I was going to be, I eventually realised I had to be much more serious than I had imagined beforehand because they (the avatars) captured the nuances so well.*

*Mark, G6: The expectation before starting was that this was going to be extremely “artificial”, but I was wrong.*

In addition, all the students took on their roles in a serious and professional manner both in the simulation and when giving peer feedback. Students consistently reported that observing peers teach in the simulation was valuable. For example, Justin mentioned that the professionalism shown by his fellow group member who enacted the teacher role, enhanced his own experience as an observer.

*Justin, G4: (…) It was exciting to observe a fellow student throwing himself into this challenge. (…) the fact that he took it so seriously elevated the experience.*

Moreover, students compared the simulated classroom to real classroom contexts. Although they raise limitations (like there being only five pupils in the classroom), they discuss key reasons why the simulation is useful nevertheless.

*Kate, G6: (…) you have had five students. I don’t know how many schools where you have classes with five students (…)*

*Kevin, G6: (Laughs)*

*Kate, G6: I don’t know. But we got to train strategies (…)*

*Kevin, G6: I agree with you. (...) we had five students in this class. But we had those five stereotypes...(...) the extreme stereotypes. And we have these in classes as well. But then, as you say, we have 20 students in between, but these student types (the avatars) are maybe those who demand extra from you as a teacher. When it comes to self-reflection, I experience that this (simulation) can prepare me for the practice period. Yes, both for practice and when starting to work. Because it’s something about stopping and thinking “what did I do now?”*

*Kate, G6: I agree. (…) If you do this a couple of times, you get the possibility to try out a whole lot of different openings, different techniques, different representations, different ways to talk, right? You get the opportunity to prepare (for real-life teaching).*

*Kevin, G6: Yes, you do. And when you in addition have the teacher educators who both give you positive feedback and suggestions afterwards… constructive feedback... it makes you more aware of the strategies you choose to use. It is very important, because, you know, it is easy to choose a track and keep to that track. The job is to challenge those habits.*

Here, Kate and Kevin discuss why they find the simulation useful both for preparing for the practicum and beyond. They acknowledge the limitation of training with only five students but conclude that repeated simulation training would benefit practice on “different openings, different techniques, different representations, different ways to talk”, as well as identifying and challenging habits. Such statements indicate how simulation training is perceived as having transformative potential. Students perceive the different possibilities for training specific skills and reflecting upon strategies.

Across most of the groups the students discussed how recognisable the interactions with the avatars were by using their prior classroom experiences as a frame of reference for discussing the authenticity of the simulation:

*Alex, G3*: *I have been a substitute several times…it’s the same feelings and things you have to think about*.

Similarly, Kate and Mark reflect upon an altercation that took place in the simulation between the avatars and how this problem situation might be dealt with.

*Kate, G6: There were obviously problems between… Ava and Ethan (two avatars). From the start, she (Ava) complained (…). No matter what he (Ethan) said, she commented! Those types of students exist*.

*Mark, G6: Such students obviously exist and that is a challenge for the classroom environment. And then you must work systematically with it and find strategies that work (…) It is not right to say “remove the behaviour” (…) but to reinforce the student’s good behaviour.*

Overall, these excerpts show at least three reasons why the simulation turns out to be believable despite the obvious constraining factors such as only having five pupils. Firstly, Sophie’s reason for taking the interaction seriously was how appropriately the nuances were captured. The human interactor controlling the avatars’ responses, makes the resulting interaction feel realistic. Secondly, Kate and Mark use previous classroom experiences as reference points when reflecting on a particular interaction between two of the avatars as a plausible encounter. Thus, the different ways of pushing back enacted by the interactor resonate as familiar. Consequently, a third reason seems to be the avatars’ archetypal personalities. The interactor uses his experience and knowledge about the scenario and the avatar personalities when aligning the degree of pedagogical and social complexity to the level of each practising student. Thus, the simulation enables students to access professionally relevant dilemmas which call for appropriate actions and reflections.

#### Technical challenges and constraining factors: “*I was fussing over the voices*”

Although most students perceive the simulation as affording meaningful practice, technical difficulties like delayed audio and inertia were consistently mentioned across the groups. In addition, students also mentioned the limitation of “*only five students*” in the simulated classroom (Samantha, G2). Students dealt with the constraints for example by “overlooking” (Pablo, G2) or by gradually becoming accustomed to the limitations:

*Sam, G4: I felt that I was struggling to take it very seriously, perhaps mostly because at first I was fussing over the voices (…) but eventually I got into it more. I still see the benefit of simulating in this way.*

Kevin (G6), referred to the avatars’ voices as somewhat *“robotic”.* One of the groups also discussed how visual graphics could be further developed for the simulation to come across as more realistic. Brian for example, who has gaming experience had expected more natural-looking graphics. However, a fellow student points out the challenge of creating realistic-looking avatars.

*Brian, G2: In the best games you are almost unable to distinguish between reality and the game (…) I am used to things being quite realistic, animation-wise.*

*Pablo, G2: When it comes to animation, it’s actually a problem to animate people, kids in particular, without making them look creepy.*

These shortcomings and dilemmas illustrate that student teachers might perceive the simulation as even more realistic if the technical issues pertaining to the voices and graphics were improved.

Lack of familiarity with the simulation was also a challenge. Although students tended to show keen interest and curiosity toward TeachLivE™, there was a need for better familiarisation with the classroom interface including knowledge about the avatars and overall the setting:

*Matthew, G4: At first I was very hung up on everything around the simulation. Was it pre-programmed, or was there one who sat and interacted with the teacher? I got curious about how it was all made. (…) I think maybe the experience could be even better if you as a teacher got more preparation time (…).*

Nonetheless, like many of his peers, Matthew was intrigued by the experience and “hung up” on how the simulation works. He mentioned needing more time to prepare. Some also suggested that it would have been beneficial to know the class beforehand, showing the need for better familiarisation. In contrast, students like for instance Alex felt well-prepared after having been through the pre-simulation briefing video stating, “*I felt like I had been to the class once before, as I watched the video” (Alex, G3)*.

#### A preferable alternative to traditional role-plays: “*So one can really just cancel physical role-plays*”

Although some students alluded to being comfortable with traditional role-plays, for the most part, they expressed that they considered the simulation less demanding. The digital puppeteering allowed them to focus on practising as professionals and not act, unlike traditional role-plays. As Sarah (G5) put it: “*I was allowed to just be myself*”. In addition to explicit statements like this, the students also speak about the simulated classroom and avatars as though they were real. This indicates that the simulation offers a space in which student teachers can embody the professional role. Kimberly (G3) suggested replacing role-plays altogether *“So one can really just cancel physical role-plays and just use this”*, implying that SBL is a feasible alternative. Such statements indicate that students perceive SBL as mediating realistic opportunities for enacting teaching. They welcome new opportunities to be able to enact practice virtually. This is of particular value for online students as further reinforced in these examples:

*Patrick, G1: Very exciting to see that virtual solutions are being worked on.*

*Justin, G4: For online students like us who study mostly over the internet, digital training can be useful.*

The students allude to needing more innovative digital instruction with an up to date “*toolbox*” (*Brian, G2)*, particularly for online teacher education.

In sum, although technical challenges and lack of familiarity were indeed constraining factors, most students perceived the simulation as affording rich learning opportunities for engaging in practice. They identify the potential in the simulation. Furthermore, the SBL design mediated a shared practice experience by enabling interaction and discourse between novices and experts. Moreover, the interaction afforded by the interactor who controls the avatars appears to be a prominent reason why most students perceived the simulation as “credible” and as representing similar personalities previously encountered in real classroom settings.

## Discussion

This study examined online initial teacher education students’ perspectives on the perceived affordances of the TeachLivE™ simulation for professional learning. The research question addressed is: What perceived affordances emerge when online student teachers engage in simulation-based learning? We have revealed five major perceived affordances which we will discuss next.

First, an educational affordance of the SBL experience was that it provided a safe learning environment for practising skills relevant to the learning outcomes, particularly pertaining to classroom management. Students referred to how the experience highlighted the importance of classroom management practices like paying attention to the social climate of the classroom. This is in line with previous findings revealing that simulations enable students to practice complex skills within conditions that provide appropriate levels of intensity (Hudson et al., 2018). Moreover, students appreciated practising without any consequences on real children, in line with previous studies (Chini et al., 2016; Spencer et al., 2019). Thus, improving teaching skills through simulation can be seen as an ethical approach (Judge et al., 2013). Unlike in real classrooms, the ability to pause the simulation for support from educators and peers and to repeat practice on targeted skills invites further educational opportunities. Students have the opportunity to hone targeted skills within a setting of reduced complexity (Grossman et al., 2009a) supported by a learning community where they receive constructive feedback. These educational affordances revealed are particularly relevant for online student teachers, who rarely have shared practice experiences with peers or teacher educators as they have fieldwork across various schools nationwide.

Our second finding pertains to social affordances. The overall SBL design yielded social interaction which was consistently appreciated by students. This involved peer and expert feedback during educator-led debriefings as well as discussions and meta-reflection during the peer-led group discussions. The social interaction elicited was based on the situated experience of enacting practice or observing peers. Studies on technology-mediated learning environments show the importance of social interaction and collaboration amongst students, as this is assumed to support learning (Kirschner et al., 2015; Kreijns et al., 2013). In addition, learning environments that enable social interaction may influence learning outcomes (Kirschner & Erkens, 2013). We found that students collaboratively discussed problems of practice and linked the simulation experiences to prior teaching experiences. They also recognised that the SBL experience was relevant to the learning outcomes and engaged in meta-reflection about dilemmas from the simulated practice. These collaborative discussions were valued and come across as a social affordance. Thus, in accordance with Kirschner et al., (2004) the properties of the learning environment invited learners to engage in social interaction. Similarly, findings by Badilla Quintana et al., (2017) showed that peer interaction was important to the SBL experience and that simulations allowed students to share practice-relevant knowledge. Furthermore, peer interactions in addition to educator-student interactions are a source of support and can be beneficial for being socialised into the profession, in turn helping students identify with the profession (Heggen & Terum, 2013). Simulations seem to be a useful tool for prompting such meaningful interactions between novices and experts. This finding is worth considering in the design of SBL interventions which often end with an educator-led debriefing. Post debriefing group discussions encourage student-led reflection and engagement over a shared practical experience. This is particularly relevant for online learning environments since online students value interactivity as important (Croxton, 2014).

Third, students had mixed reactions regarding the authenticity of the simulation, for instance, they expressed curiosity, surprise, doubt and critique. These aspects pertain to the usability of the simulation. Nonetheless, in addition to explicit statements about realism, we also find implicit indicators of perceived authenticity. Similarly, Dalinger et al., (2020) found that while most of the participants perceived the simulation as authentic, some were startled by the spontaneous nature of the avatar interactions and others found it challenging to completely suspend disbelief. Our findings suggest that the real-time interaction, afforded by the interactor who through natural, convincing responses helps bring the experience to life. This makes TeachLivE™ a useful artefact for supporting professional learning.

Fourth, technical challenges and lack of familiarity were perceived by students as constraining factors. This is in line with previous research on TeachLivE™ (Dalinger et al., 2020; Judge et al., 2013). In our findings, students commented on the “robotic” voices and inertia. Moreover, some had expected sophisticated graphics to attain a sense of realism, as mentioned by a student with gaming experience. One explicitly acknowledged the problem of developing human-like avatars, stating that they could appear “creepy”. Still, most seemed focused on the realistic classroom interaction as an indicator of authenticity. To this end, we concur with Howard (2017) who argues that the uncanny valley effect should be considered in research on simulations. The uncanny valley refers to the sense of uneasiness felt by participants when animatronics cannot be distinguished from humans (Howard, 2017). Thus, although digital artefacts may afford new possibilities, they also introduce new sets of challenges (Wertsch, 1998).

In addition to technical challenges, students reported needing better familiarisation with the technology. This shows the importance of a detailed briefing phase for students to be better acquainted with the technology. Also, having the possibility to practice several times in the classroom simulation would both help with preparedness and familiarity. This would also possibly reduce the nervousness that may be associated with being observed by peers, educators and researchers. Walters et al., (2021) who conducted a pretest-posttest control group study also found that students who practised in the simulation were more nervous before the session in comparison to traditional role-plays. However, the students showed less worry over subsequent sessions (Bautista & Boone, 2015; Walters et al., 2021) given the benefits of repeated practice. While having additional practice sessions would be beneficial, one major barrier to the integration of SBL in teacher education is the high cost of the simulation including the cost of the interactor. This is rarely reported in the literature (Bondie et al., 2021) and has implications for how scalable SBL is in teacher education.

Our final finding reveals that students preferred simulation-based training over role-plays. Despite the technological challenges, overall the simulation is generally perceived as useful. Unlike traditional role-plays, the simulation enabled students to either teach or observe without the demanding task of acting since this was accounted for by the interactor. The experienced interactor afforded students robust training using his professional experience to respond appropriately. Leveraging the benefits of desktop simulations over traditional role-plays can also be seen as potentially transforming typical practices. Previous research shows the efficacy of simulations over traditional role-play methods. For instance, in a randomised control study comparing typical practice with simulation training, Walters et al., (2021) found that students who practised in the simulation viewed it as a strong means for professional practice, compared to traditional live practice.

Overall, the vast majority of students perceive TeachLivE™ as providing opportunities for practising skills such as classroom management based on plausible, professionally relevant scenarios. Although there were mixed sentiments regarding the realism of the simulation, the simulation mediated both practice and social interaction. Thus, consistent with the sociocultural notion that artefacts mediate learning and social interaction, our findings support the notion that simulations mediate professional learning (Orland-Barak & Maskit, 2017). In particular, we argue that TeachLivE™ mediates professional learning through the interactor who facilitates teacher-avatar interactions which are “authentic, relevant, and deal with real-life problem-solving situations and management of dilemmas” (Orland-Barak & Maskit, 2017, p. 2). Thus a perceived affordance of TeachLivE™ is that it enables additional practice and reflection opportunities in safe frames, thereby having transformative potential in university teacher education.

## Limitations and future directions

We have provided rich qualitative data to report online students’ perspectives on simulation-based learning, however, our findings should be considered in light of several limitations. Our findings are based on one SBL intervention within one specific setting, thus limiting generalisability. However, following Tracy (2010) we have provided rich description which may invite transferability (Lincoln & Guba, 1985) to similar contexts. Moreover, because the intervention was about students’ initial experience with simulation-based learning, we cannot claim that the course learning outcomes were reached.

Through this research, we have seen that there is a need to gain deeper insight into the actual core practices that students pay attention to during the simulation and the debriefing. Thus, future research can aim at investigating how simulations may support students’ ability to professionally notice and attend to salient events concerning core practices in teacher education. In addition, although we did not focus on gender in this study, we observed that significantly more males opted to teach in the simulation and that those who were most engaged in the discussions about avatar graphics and voices had gaming experience. Thus, future research could also examine student engagement in SBL in relation to gender and previous gaming experience.

## Conclusion

In this study, we examined online students’ perspectives on the affordances of simulation-based learning. Through the simulation, the students either engaged in the simulation or observed and prepared peer feedback. During the educator-led debriefing, students learned to give and receive professional feedback, while in peer-led group discussions they collaboratively reflected, linking the simulation experience to the learning outcomes and work-life situations. Given the traditional theoretical focus in teacher education, integrating SBL in this manner could influence established, culturally-rooted practices. In so doing, students are provided with shared practical experiences which students in general have lacked and online students have a particular need for. Our data shows specifically that the simulation afforded practical experience with skills such as classroom management. Thus, it is possible that integration of such SBL interventions for classroom management training may in turn prevent the attrition of early career teachers.

## Electronic supplementary material

Below is the link to the electronic supplementary material.

**Additional file 1**: Appendix A

## Data Availability

The anonymised data used for the analysis of the current study can be obtained from the corresponding author.

## Footnotes

4 This data is part of a larger data set including the actual interactions with the avatars followed by the educator-led debriefings. Video-recorded observations from the actual interactions that took place in the simulation as well as the contents of the debriefing will be analysed and discussed in our future research.

5 Questions that guided the peer-led focus groups and written reflections are attached as Appendix A.

6 Nvivo.

## List of abbreviations

Simulation-based learning (SBL).
