# Supplementary material for: The perceived affordances of simulation-based learning: online student teachers’ perspectives
Source: Int J Educ Technol High Educ. 2022 Dec 19;19(1):60. doi: 10.1186/s41239-022-00366-2 (PMC9760319; doi:10.1186/s41239-022-00366-2)
Supplement: Supplementary file 1 — Additional file 1: Appendix A [file 41239_2022_366_MOESM1_ESM.docx]

# **Appendix A**

Time frame: 45 minutes

## **Written reflection**

*Spend up to 15 minutes writing a reflection note:*

1. Were you an observer or teacher?
2. What was the focus of your group? Mathematics didactics or Pedagogy?
3. How do you describe your first impression of the simulation?
4. Decide on the following statements: TeachLivE strengthens my knowledge of pedagogy / mathematics didactics?

| 1 = strongly disagree | 2 = disagree | 3= neither agree nor disagree | 4 = agree | 5 = strongly agree |
| --- | --- | --- | --- | --- |

Explain your answer:

1. Did you experience anything else? Explain.
2. Other comments?

## **Peer-led Focus Group Discussions**

*Spend up to 30 minutes discussing:*

1. What competencies did you train in the simulation? * (see learning objectives A or B)
2. Did you experience that the simulation training helps to prepare you for practice? Explain.
3. Did you “believe” in the situation? Did you feel that what happened seemed authentic/credible?
4. Do you have previous experience with role-playing in teacher education? If so, do you see any advantages or disadvantages to practising the role of a teacher in a digital simulation compared to a traditional role-playing game?
5. Would it be appropriate to practice several times on similar simulations (not instead of practice, but in addition)? Yes/ No?
6. To what extent and in what ways do the group discussions (both with and without a teacher) have an impact on the learning outcomes? (see learning objectives A or B)

**Relevant learning outcomes (A. Mathematics or B. Pedagogy and student knowledge)**

**A. Learning Outcomes MG2MA1 (Mathematics)**

Candidates:

- have knowledge of concretisation and representation of mathematical concepts and of the significance this has for the progression in students' learning in the process from concrete to abstract,
- have knowledge of different representations and of the significance that the use of and transitions between representations can have for students' learning,
- can analyse and evaluate students' ways of thinking, argumentation and solution methods,
- can use working methods that promote students' wonder, creativity and ability to work systematically with exploratory activities, reasoning and argumentation.

**B. Learning Outcomes MG2PE3 and MG2PE4 (Pedagogy and student knowledge)**

Candidates:

- have knowledge of how teachers should face learning difficulties and adaptation and behavioral challenges in children and young people in grades 5-10,
- based on knowledge of social systems, can critically analyse interactions in classes and groups of students and make decisions that stimulate students' learning,
- can analyse social structures and make decisions that promote the student as an actor in their psychosocial environment,
- can facilitate interaction in classes and grades 5.-10. and can critically analyse the activity,
- can communicate with students and parents in a way that promotes cooperation and understanding regardless of culture, background, religion or gender.
